# Health-Related Quality of Life and Its Correlation With Depression Among Chinese Centenarians

**DOI:** 10.3389/fpubh.2020.580757

**Published:** 2020-10-30

**Authors:** Ke Han, Shanshan Yang, Wangping Jia, Shengshu Wang, Yang Song, Wenzhe Cao, Jianwei Wang, Miao Liu, Yao He

**Affiliations:** ^1^State Key Laboratory of Kidney Disease, Beijing Key Laboratory of Aging and Geriatrics, National Clinical Research Center for Geriatrics Diseases, Institute of Geriatrics, The 2nd Medical Center of Chinese PLA General Hospital, Beijing, China; ^2^Department of Disease Prevention and Control, The 1st Medical Center, Chinese PLA General Hospital, Beijing, China; ^3^Department of Statistics and Epidemiology, Graduate School, Chinese PLA General Hospital, Beijing, China

**Keywords:** centenarians, depression, comorbidity, chronic disease, health-related quality of life

## Abstract

**Background:** As evidence on depression and health-related quality of life (HRQoL) among the oldest-old is currently limited, this study aimed to re-examine the association between depression and HRQoL among centenarians.

**Methods:** We analyzed cross-sectional data from the China Hainan Centenarian Cohort Study (CHCCS). The 15-item Geriatric Depression Scale (GDS-15) and three-level EuroQol five-dimensions (EQ-5D-3L) were used to evaluate depression and HRQoL, respectively. Poor health states were defined as EQ-5D index <0.665. Based on their GDS-15 score, individuals were categorized into three stages of depression: major depressive disorder (MDD; score ≥10), minor depressive disorder (MnDD; score between 6 and 9), and normal (score ≤ 5). Based on sex and comorbidity stratification, multivariable logistic regression was used to calculate the risk of poor health state in different levels of depression. We also used restricted cubic splines with a knot at 5 points (GDS-15) to flexibly model the association of GDS-15 scores with poor health states.

**Results:** Totally, 1,002 participants were included in this study for analysis. Participants' median age was 102 years, and 82.04% were female. The median EQ-5D index was 0.68 (range: −0.149–1), and the mean VAS and GDS-15 scores were 61.60 (range: 0–100), and 5.23 (range: 0–15), respectively. Centenarians with MnDD and MDD accounted for 38.12 and 9.98%, respectively. While those with poor health states accounted for 45.11%. For every 1-point increase in GDS-15, the risk of poor health state increased by 20% (*P* < 0.001) after an adjustment for age, gender, ethnicity, marital status, education, residence type, smoking, drinking, weekly exercise, body mass index category, serum albumin, 25-hydroxyvitamin D, C-reactive protein, and comorbidities. MnDD and MDD were independent risk factors for poor health state (MnDD, OR = 2.76, *P* < 0.001; MDD, OR = 3.14, *P* < 0.001). The association was more prominent in centenarians without comorbidity.

**Conclusions:** This study demonstrated a negative association between depression and HRQoL in Chinese centenarians, especially in centenarians without comorbidity. Large-scale prospective studies are needed to corroborate our findings and provide more information about the causal inference and internal mechanisms of this association.

## Introduction

The global population is aging. Worldwide, the proportion of the population aged 60 years and older increased from 9.2% in 1990 to 11.7% in 2013, and will continue to increase and account for 21.1% of the world's population by 2050 (1). Due to dramatic changes in population structure, aging-related problems have significantly impacted social and economic development, health and disease prevalence patterns, and individuals' lifestyles.

In recent years, there has been an increased emphasis on promoting healthy aging. Health-related quality of life (HRQoL) is a widely used instrument to evaluate individuals' daily activities, physiological functions, and subjective satisfaction in their emotional and social life (2). HRQoL comprehensively reflects the health-related factors of older adults, covering all aspects of the biopsychosocial model; it is considered an important indicator of healthy aging (3). As centenarians are considered an ideal template for healthy aging (4–6), research on this population may contribute to an enhanced understanding of the mechanisms and determinants of healthy aging.

Depression is the most common mental disorder in older adults (7). Especially in the oldest-old age group, depression is more prevalent and complicated due to age-related risk factors such as disease and functional decline (8–10). In a study of older adults over 90 years of age, the prevalence of depression was related to an overall decreased health status and quality of life and with increased mortality (11). The relationship between depression and HRQoL has been demonstrated in the general older population (10, 12) and people with certain diseases such as diabetes (13), breast cancer (14), sensory disabilities (15), and HIV (13–16). However, no previous study has investigated the association in centenarians. In consideration of the age-related vulnerabilities and potential unique patterns related to longevity (17, 18), there is a need to examine the association between depression and HRQoL in the oldest-old.

Therefore, we assessed the baseline characteristics of HRQoL and investigated the association with the levels of depressive disorder, especially the difference in sex and comorbidities, using cross-sectional data obtained from a complete sample of a centenarian cohort from regions in China with the oldest population.

## Methods

### Study Population

This study derived information from the baseline data of China Hainan Centenarian Cohort Study (CHCCS). Hainan Province has the highest percentage of centenarians (18.75/100,000) and average life expectancy (76.3 years) in China (19, 20). Furthermore, as a relatively closed island area, the low proportion of immigrants ensured a high homogeneity of centenarians. We adopted a longitudinal observational design based on a complete sample of both community-dwelling and institutionalized population in Hainan, China. CHCCS is a complete sample study involving all centenarians in Hainan Province. The sampling frame and investigation methods of CHCCS are outlined in previous reports (21). This study was approved by the Ethics Review Committee of the People Liberation Army General Hospital in Beijing, China, and written informed consent was obtained from all participants.

A baseline survey was conducted from June 2014 to December 2016. In 2014, there were 1,811 living centenarians in total, according to the household register. After a rigorous validation, we excluded the people with wrong registration information (*n* = 18), residential address mismatches (*n* = 55), age mismatches (*n* = 58), and who had died (*n* = 207). Totally, 1,473 eligible centenarians were identified from the Civil Affairs Bureau of Hainan Province in 2014. Excluding lost interviews due to inability to complete the investigation (*n* = 124), death before interview (*n* = 268), and refusal to participate (*n* = 79), 1,002 centenarians were included for analysis.

### Data Collection

The baseline survey mainly included a questionnaire interview, physical examination, biological specimen collection, and laboratory examination. All the questionnaires were conducted through face-to-face interviews by the systematically trained nurses in the Hainan dialect. The content of the questionnaire survey included general demographic characteristics, lifestyles, personal, and family disease history, cognitive and mental health status, and functional status. Questionnaire items that participants were unable to answer or self-assessed were answered by their closest caregivers. Epidata 3.1 software was used for data entry, and all data were cross-checked by two researchers.

### Depression and Depressive Disorders

We used the 15-item Geriatric Depression Scale (GDS-15) to measure depression in centenarians (22). The scale assesses the depression status of participants since the last week, mainly testing older adults' feelings of depression, reduced activity, irritability, withdrawal and pain, and negative views of the past, present, and future. There are 15 items on this scale, and each item requires the participants to answer “yes” or “no.” Each answer indicating depression counts 1 point, with a maximum score of 15 points. The higher the score, the more obvious the symptoms of depression. Depressive disorders were defined using the cutoff points for community-dwelling elders (23): ≤ 5, normal; 6–9, minor depressive disorder (MnDD); ≥10, major depressive disorder (MDD).

### Health-Related Quality of Life

HRQoL was measured using the three-level EuroQol five-dimensions (EQ-5D-3L), which is a general tool for describing and evaluating health states (24, 25). The EQ-5D-3L covers three levels of the five dimensions: mobility, self-care, daily activities, pain/discomfort, and anxiety/depression. To quantify participants' preferences, a time trade-off (TTO) model based on a specific population was used to calculate the EQ-5D index. The EQ-5D index was derived from the Chinese EQ-5D-3L value set (26), and it ranged from −0.149 to 1. An EQ-5D index of 1 indicated full health in all 5 dimensions. Under this TTO value set, mild states were health states in which dimensions were either in level 1 (no problems) or in level 2 (some/moderate problems) and with a maximum of 3 dimensions impaired, which means that EQ-5D index ≥0.665 (excluding “full health”). Therefore, we defined poor health states as EQ-5D index <0.665, and normal health states as EQ-5D index ≥0.665 in this study. The Visual Analog Scale (VAS) is a self-assessment tool for participants to assess their health status on a 20 cm vertical visual scale with a grade ranging from 0 (worst imaginable health state) to 100 (best imaginable health state).

### Covariates

Socio-demographic characteristics assessed included age, sex, education, ethnicity, marital status, education, and residence type at the time of face-to-face interviews. We also considered lifestyle characteristics such as smoking status, alcohol drinking status, and weekly exercise. Some important indicators including body mass index (BMI), serum albumin (Alb), 25-hydroxyvitamin D[25 (OH)D], and C-reactive protein (CRP) were also considered for analysis. Participants were asked to report whether they had been diagnosed and treated by a doctor for any specific medical conditions. The presence of heart disease, stroke, chronic obstructive pulmonary disease, and cancer were self-reported. The presence of hypertension was defined by a self-report of high blood pressure, and/or sitting systolic blood pressure >140 mmHg and/or diastolic blood pressure >90 mmHg (27). Similarly, diabetes was defined by a self-report and/or a fasting blood glucose concentration of ≥7.0 mmol/L (28). Chronic renal dysfunction was defined by a self-report and/or a glomerular filtration rate <60 ml·min-1/1.73 m^2^ (29). In this study, comorbidities were defined as two simultaneously occurring chronic diseases in addition to depression and poor health states. The process of clinical examination and biological specimen collection were outlined in previous reports (21).

### Statistical Analyses

All continuous variables were assessed by QQ plot and Shapiro–Wilk normality test. Normally distributed continuous variables were expressed as mean ± standard deviation (SD); non-normal continuous variables were expressed as median (interquartile range [IQR]); and categorical variables were presented by counts and percentages. The differences between the means (medians) and proportions of the two groups were compared by Student's *t*-test (Wilcoxon rank-sum test) and chi-square test. We applied logistic regression analysis to calculate the odds ratios (ORs) of poor health states, using the continuous and categorical forms of GDS-15 as independent variables. Due to the uneven distribution and different vulnerabilities of depression and impaired health status between men and women, we conducted a stratification analysis on gender. In addition, we observed the association between depression and HRQoL according to the comorbidity category. Further, we tested the interactions of comorbidities and GDS-15 categories on HRQoL in different models. In order to avoid the influence of subjectivity and information loss for the number of categories and node positions in the classification, we also used restricted cubic splines with five knots at the 5th, 35th, 50th, 65th, and 95th centiles to flexibly model the association of GDS-15 scores with poor health states and examine their non-linear associations. In multivariable analyses, model 1 were adjusted for age, gender, ethnicity, marital status, education, and residence type, and model 2 were additionally adjusted for smoking, drinking, weekly exercise, BMI category, serum albumin, 25-hydroxyvitamin D, C-reactive protein, and comorbidities. Demographic characteristics and lifestyle-related variables were directly included into the models, and covariates that met any of the following criteria were included in the fully adjusted model: (1) the inclusion of covariates in the basic model or the elimination of covariates from the complete model has an impact on the regression coefficient of >10%; (2) *p*-value of the regression coefficient between the covariate (X) and dependent variable (Y) was <0.1. All statistical analyses were performed using SPSS Statistics version 24.0 (IBM Corporation, Armonk, NY, United States) and Empower Stats (X&Y Solutions, Inc., Boston, MA). A *p* < 0.05 (2-tailed) was considered statistically significant.

## Results

### Baseline Characteristics

Table 1 summarizes the general characteristics of the 1,002 participants (180 men and 822 women) by the categories of HRQoL. Participants' ages ranged from 100 to 116 years, with a median age of 102 years (IQR, 101–104). The median EQ-5D index was 0.68 (IQR, 0.55–0.79; range: −0.149–1.000) and the average VAS score was 61.60 ± 15.56 (range: 0–100). Among the 1,002 participants, 452 centenarians (66 men and 386 women) reported a poor health state, accounting for 45.11%. Totally, 38.12% of participants had MnDD and 9.98% of participants had MDD. Compared with participants in normal health states, participants in poor health states had a significantly lower GDS-15, VAS, and EQ-5D index, and a higher proportion of depressive disorders. Significant differences were also found between the two groups in terms of sex, residence type, alcohol drinking, weekly exercise, Alb, 25(OH)D, CRP, and BMI categories.

**Table 1:** General characteristics of 1,002 centenarians according to the HRQoL categories^a^^,^^b^.

**Characteristics**	**Total(*n* = 1,002)**	**Normal HRQoL (*n* = 550)**	**Poor health state (*n* = 452)**	***P*-value**
Age, year	102.00 (101.00–104.00)	102.00 (101.00–104.00)	102.00 (101.00–104.00)	0.224^*^
GDS-15	5.23 ± 3.05	4.42 ± 2.86	6.23 ± 2.97	<0.001
VAS	61.60 ± 15.56	66.35 ± 13.78	55.82 ± 15.67	<0.001
EQ-5D index score	0.68 (0.55–0.79)	0.79 (0.68–0.89)	0.50 (0.30–0.59)	<0.001^*^
Alb, g/L	38.43 ± 3.99	39.42 ± 3.49	37.22 ± 4.23	<0.001
CRP, mg/dl	0.21 (0.08–0.58)	0.17 (0.07–0.49)	0.29 (0.09–0.58)	<0.001^*^
25(OH)D, ng/mL	22.74 ± 9.24	24.00 ± 9.06	21.22 ± 9.24	<0.001
**Depression**				<0.001
Normal	520 (51.90%)	353 (64.18%)	167 (36.95%)	
MnDD	382 (38.12%)	163 (29.64%)	219 (48.45%)	
MDD	100 (9.98%)	34 (6.18%)	66 (14.60%)	
**Gender**				0.012
Male	180 (17.96%)	114 (20.73%)	66 (14.60%)	
Female	822 (82.04%)	436 (79.27%)	386 (85.40%)	
**Ethnicity**				0.796
Han	883 (88.12%)	486 (88.36%)	397 (87.83%)	
Others	119 (11.88%)	64 (11.64%)	55 (12.17%)	
**Education**				0.071
Illiterate	915 (91.32%)	493 (89.64%)	422 (93.36%)	
Primary school	67 (6.69%)	42 (7.64%)	25 (5.53%)	
Middle school or higher	20 (2.00%)	15 (2.73%)	5 (1.11%)	
**Marital status**				0.384
Married	100 (9.98%)	59 (10.73%)	41 (9.07%)	
Widowed/ divorced/ never married	902 (90.02%)	491 (89.27%)	411 (90.93%)	
**Residential type**				0.002
Living together with families	863 (86.13%)	457 (83.09%)	406 (89.82%)	
Living alone at home	139 (13.87%)	93 (16.91%)	46 (10.18%)	
**BMI categories**				<0.001
BMI <18.5kg/m^2^	575 (57.39%)	286 (52.00%)	289 (63.94%)	
18.5 ≤ BMI <24 kg/m^2^	393 (39.22%)	242 (44.00%)	151 (33.41%)	
BMI≥24kg/m^2^	34 (3.39%)	22 (4.00%)	12 (2.65%)	
**Smoking status**				0.794
Non-smoker	893 (89.12%)	492 (89.45%)	401 (88.72%)	
Former	74 (7.39%)	38 (6.91%)	36 (7.96%)	
Current	35 (3.49%)	20 (3.64%)	15 (3.32%)	
**Alcohol drinking**				0.02
Non-drinker	824 (82.24%)	444 (80.73%)	380 (84.07%)	
Former	79 (7.88%)	39 (7.09%)	40 (8.85%)	
Current	99 (9.88%)	67 (12.18%)	32 (7.08%)	
**Weekly exercise**				<0.001
Yes	129 (12.87%)	119 (21.64%)	10 (2.21%)	
No	873 (87.13%)	431 (78.36%)	442 (97.79%)	
**Comorbidity**				0.755
Yes	331 (33.03%)	184 (33.45%)	147 (32.52%)	
No	671 (66.97%)	366 (66.55%)	305 (67.48%)	

*HRQoL, health-related quality of life; EQ-5D, EuroQol five dimensions questionnaire; GDS-15, 15-item Geriatric Depression Scale; VAS, Visual Analog Scale; Alb, serum albumin; 25(OH)D, 25-hydroxyvitamin D; CRP, C-reactive protein; MnDD, minor depressive disorder; MDD, major depressive disorder; BMI, body mass index*.

a *Normally distributed continuous variables were expressed as mean ± standard deviation (SD); non-normal continuous variables were expressed as median (interquartile range); categorical variables were presented by the percentage*.

b *Differences between two groups were evaluated by t-test or chi-square test*.

* *Differences between two groups were evaluated by Wilcoxon rank-sum test or Fisher's exact chi-square test*.

### Association of GDS-15 and Depressive Disorders With Poor Health States

As shown in Table 2, continuous and categorical forms of GDS-15 were used as independent variables and the dichotomous EQ-5D index as the dependent variable. The demographic characteristics, lifestyle, and other covariates [BMI category, Alb, 25(OH)D, CRP, and comorbidities] were gradually adjusted, and multiple logistic regression analyses were performed. In the total study population, for every 1-point increase in GDS-15, the risk of poor health state increased by 24, 25, and 20% in each model (*P* < 0.001). Similar results were found in men and women.

**Table 2:** Odds ratios for poor health states among centenarians with different levels of depression^a^^,^^b^.

	**GDS-15 score**	**Categorized variables of GDS-15 score**			***P* for trend**
		**Normal**	**MnDD**	**MDD**	
**Total**
Crude model	1.24 (1.18, 1.29) <0.001	Ref.	2.84 (2.16, 3.73) <0.001	4.10 (2.61, 6.45) <0.001	<0.001
Model 1	1.25 (1.19, 1.31) <0.001	Ref.	2.84 (2.14, 3.76) <0.001	4.42 (2.76, 7.07) <0.001	<0.001
Model 2	1.20 (1.14, 1.27) <0.001	Ref.	2.76 (2.03, 3.76) <0.001	3.14 (1.90, 5.20) <0.001	<0.001
**Male**
Crude model	1.27 (1.12, 1.44) 0.001	Ref.	3.91 (1.98, 7.72) <0.001	3.62 (0.91, 14.39) 0.067	0.001
Model 1	1.27 (1.11, 1.45) <0.001	Ref.	3.79 (1.83, 7.87) <0.001	2.90 (0.67, 12.46) 0.152	0.005
Model 2	1.23 (1.06, 1.42) 0.008	Ref.	3.62 (1.59, 8.25) 0.002	1.83 (0.36, 9.27) 0.464	0.044
**Female**
Crude model	1.22 (1.16, 1.29) <0.001	Ref.	2.61 (1.93, 3.52) <0.001	3.95 (2.44, 6.40) <0.001	<0.001
Model 1	1.25 (1.18, 1.31) <0.001	Ref.	2.64 (1.93, 3.60) <0.001	4.47 (2.70, 7.38) <0.001	<0.001
Model 2	1.21 (1.14, 1.28) <0.001	Ref.	2.61 (1.85, 3.68) <0.001	3.47 (2.01, 6.00) <0.001	<0.001

a *Model 1: Adjusted for age, gender, ethnicity, marital status, education, and residence type. Model 2: Adjusted for age, gender, ethnicity, marital status, education, and residence type, smoking, drinking, weekly exercise, BMI category, serum albumin, 25-hydroxyvitamin D, C-reactive protein, and comorbidities*.

b *Data are represented as OR (95% CI) P-value*.

Compared with the normal group of depression, the multivariable logistic analysis revealed a significant association between depressive disorders and poor health states in total population (Model 2: MnDD, OR = 2.76, *P* < 0.001; MDD, OR = 3.14, *P* < 0.001). The risk of MnDD in male centenarians [Model 2, OR = 3.62, 95% confidence interval (CI):1.59–8.25, *P* = 0.002] was higher than in female centenarians (Model 2, OR = 2.61, 95% CI: 1.85–3.68, *P* < 0.001). The risk of MDD was 2.47 times higher than the normal group of depression in female centenarians (Model 2, OR = 3.47, 95% CI: 2.01–6.00, *P* < 0.001), but not significant in male centenarians (Model 2, OR = 1.83, 95% CI: 0.36–9.27, *P* = 0.464). Furthermore, it was observed in all models that the health states deteriorated with the severity of depression (*P* for trend <0.05).

**Figure 1:**
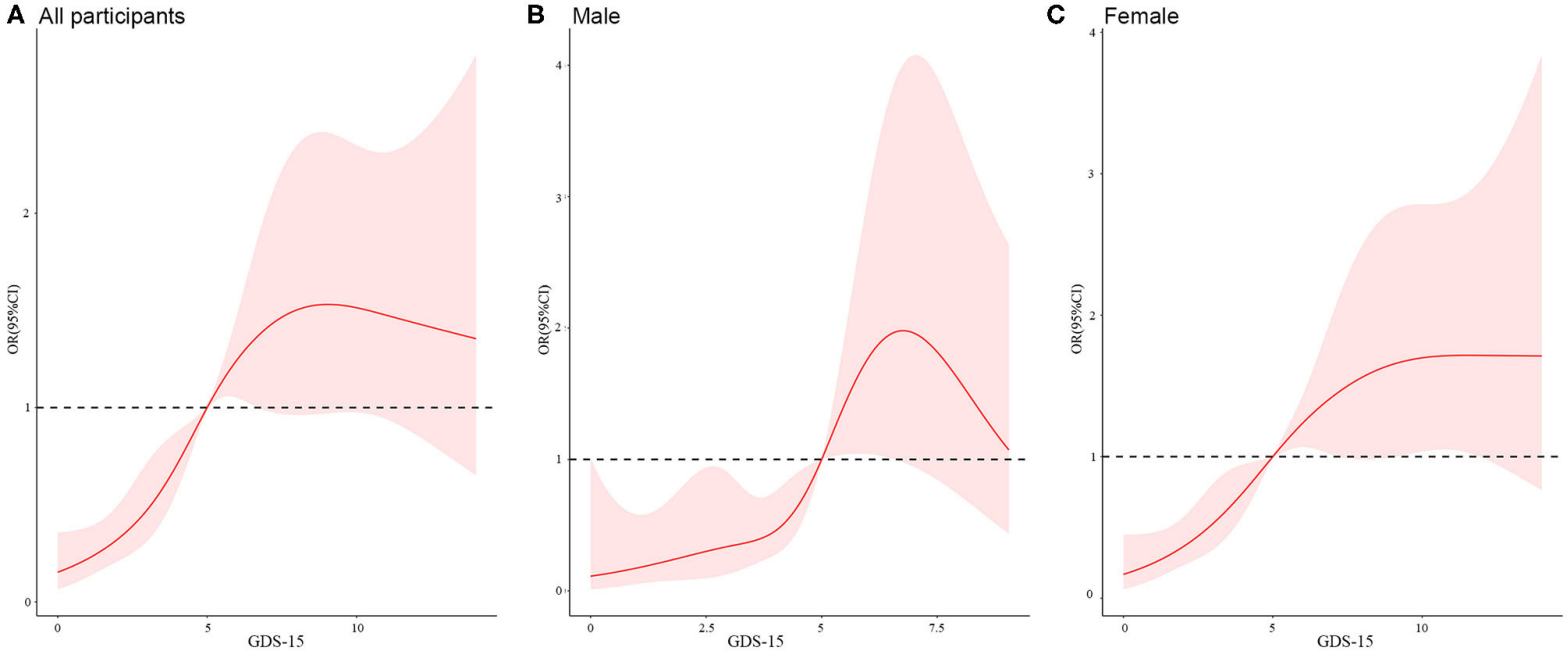
Restricted cubic splines of the relationship between poor health states and GDS-15 score^a,b^ in the total study population **(A)**, male population **(B)**, and female population **(C)**. ^a^Adjusted for age, gender, ethnicity, marital status, education, and residence type, smoking, drinking, weekly exercise, BMI category, serum albumin, 25-hydroxyvitamin D, C-reactive protein, and comorbidities. ^b^The GDS-15 score of 5 was set as the reference point (OR = 1).

In Figure 1, we used restricted cubic splines to flexibly model and visualize the relation of GDS-15 scores with poor health states. We have observed that there is a non-linear relationship between the GDS scores and poor health states of male centenarians, female centenarians, and all participants (*P* = 0.010, 0.014, and <0.001, respectively). We have observed an S-type association of GDS-15 scores with poor health states that among female centenarians, the risk of poor health states increased as the GDS-15 score increases, but the higher the score, the increase gradually slowed compared to the reference point (GDS-15 = 5). However, it has not been observed that the growth in GDS scores was related to the improvement of HRQoL at high levels of GDS scores.

### Comorbidity-Stratified Analyses

The associations of GDS-15 and depressive disorders with poor health states in centenarians with and without comorbidities were explored (Table 3). In all models, we found that in the group with comorbidities, the increased risk of poor health states due to an increase in GDS-15 was lower than that in the group without comorbidities. In centenarians with comorbidities, compared with normal group, participants with MnDD, and MDD had a 1.88- and 0.56-fold increased risk of poor health state in the fully adjusted model, but this association was not statistically significant in MDD (MnDD: OR = 2.88, 95% CI: 1.61–5.14, *P* < 0.001; MDD: OR = 1.56, 95% CI: 0.63–3.86, *P* = 0.340). Interestingly, among centenarians who did not suffer from comorbidities, the association between depressive disorders and poor health states risk was more prominent (MnDD: OR = 2.98, 95% CI: 2.03–4.37, *P* < 0.001; MDD: OR = 5.09, 95% CI: 2.66–9.72, *P* < 0.001). The *p*-values of the interaction test of comorbidities and depression on HRQoL in the crude model, Models 1 and 2 were 0.026, 0.017, and 0.059, respectively.

**Table 3:** Odds ratios for poor health states among centenarians with different levels of depression with or without comorbidities^a^^,^^b^.

	**Continuous variable of GDS-15 score**	**Categorized variables of GDS-15 score**			***P* for trend**	***P* for interaction c**
		**Normal**	**MnDD**	**MDD**		
**With comorbidity**
Crude model	1.15 (1.07, 1.25) <0.001	Ref.	2.73 (1.69, 4.43) <0.001	1.75 (0.81, 3.76) 0.153	0.01	0.026
Model 1	1.17 (1.08, 1.28) <0.001	Ref.	2.92 (1.73, 4.93) <0.001	1.93 (0.85, 4.38) 0.118	0.009	0.017
Model 2	1.16 (1.06, 1.27) 0.002	Ref.	2.88 (1.61, 5.14) <0.001	1.56 (0.63, 3.86) 0.340	0.05	0.059
**Without comorbidity**
Crude model	1.28 (1.21, 1.36) <0.001	Ref.	2.95 (2.11, 4.13) <0.001	6.45 (3.59, 11.59) <0.001	<0.001	
Model 1	1.30 (1.22, 1.38) <0.001	Ref.	2.95 (2.09, 4.16) <0.001	7.00 (3.83, 12.80) <0.001	<0.001	
Model 2	1.25 (1.17, 1.33) <0.001	Ref.	2.98 (2.03, 4.37) <0.001	5.09 (2.66, 9.72) <0.001	<0.001	

a *Model 1: Adjusted for age, gender, ethnicity, marital status, education, and residence type. Model 2: Adjusted for age, gender, ethnicity, marital status, education, and residence type, smoking, drinking, weekly exercise, BMI category, serum albumin, 25-hydroxyvitamin D, C-reactive protein, and comorbidities*.

b *Data are represented as OR (95% CI) P-value*.

c *The interactions of comorbidities and GDS-15 categories on HRQoL were tested in different models*.

## Discussion

To the best of our knowledge, this is the first study focused on the association between depression and HRQoL in a population-based sample of centenarians in China. We found that the elevated depression levels and the presence of depressive disorders were associated with the decrease in HRQoL, and this association was more prominent in centenarians without comorbidity. Therefore, this study provides evidence of the relationship between depression and HRQoL in the oldest-old and new information about comorbidities.

A study about EQ-5D-5L norms for the urban Chinese population in China has reported that 54% of the sample reported their health as “perfect health,” and the average EQ-5D scores of men and women in the age group older than 70 years were 0.932 (SD: 0.034) and 0.912 (SD: 0.031), respectively (30). A Hong Kong survey of adults with an average age of 72.74 years showed that the participants' average EQ-5D index was 0.83 (31). Another longitudinal study reported that 17% of the older adults in northern Italy was perfectly health in HRQoL (32). The HRQoL level of centenarians in the CHCCS was worse than these studies. This may be due to the differences in age groups and the representativeness of the participants. A study based on the Spanish population showed that the norms for the EQ-5D index and VAS in the age group of 85 years and higher were 0.622 (95% CI, 0.591–0.652) and 54.6 (95% CI, 52.4–56.7) (33), which is lower than those in our study. This may indicate that the self-reported health status of the centenarians in this study was better than the reported population.

In this study, both depression levels and depressive disorders were found to have a negative correlation with HRQoL. These results are in line with previous studies conducted on the general older adult population (10). A cohort study that included individuals over 90 reported that the presence of depression was associated with a decline in overall functional status, a decline in HRQoL, and increased mortality (11). In addition, in studies of people with certain diseases such as diabetes (34), breast cancer (14), and HIV (16), depression was negatively associated with HRQoL. Our findings contribute new evidence in centenarians and may contribute to a better understanding of the determinants of improving the HRQoL. However, the causality between depression and HRQoL remains uncertain. In a representative survey of the German general population aged 75 years and over (12), it was found that there was a significant negative correlation between the initial changes in HRQoL and the subsequent changes in GDS-15, and not conversely. This conflicts with the findings of Van der Weele (11). Thus, this study is insufficient to solve the above controversy, and follow-up cohort studies are needed to explore the direction of development between depression and HRQoL.

In the fully adjusted model, MDD in male centenarians was not significantly associated with poor health states (*P* = 0.464). Considering the direction of association (OR = 1.83, 95% CI: 0.36, 9.27) and results of continuous variables of GDS-15 (Model 2, OR = 1.23, 95% CI: 1.06, 1.42, *P* = 0.008), this may be due to the insufficient sample size of the male centenarians, and may not be a reflection of the actual correlation between depression and poor health states in this population. However, we have not found any other research evidence to prove our results. A previous meta-analysis using nationally representative samples demonstrated that gender differences exist in depression symptoms throughout life (35). As for the prevalence of depression, women are almost twice as likely to experience depression than men across the lifespan (36). On the other hand, older men and women may have different perceptions of HRQoL, and women are more likely to report worse HRQoL (37). The susceptibility of biological and psychological aspects between different sexes and the influence of environmental factors at the macro and micro levels make the association complicated. But even among centenarians of different genders, we should pay more attention to the oldest-old with severe depressive disorder, because they are more likely to be accompanied by a low level of the HRQoL.

It is somewhat surprising that in centenarians with comorbidities, the increased risk of poor health state due to elevated GDS-15 was lower than those without comorbidities. It is generally considered that multimorbidity aggravates both depression (7) and HRQoL (38) in older individuals. Studies based on the Chinese population found that participants with chronic diseases had a significantly lower EQ-5D index than participants without diseases (39), and the impact of comorbidities on HRQoL changed due to different disease combinations (40). There may be two explanations for our results. With aging, the older may gradually accept the decline in physical function and the deterioration in health due to changes in biology and social psychology, which may change their internal standards of health and reduce expectations (10). Therefore, HRQoL of centenarians may score higher even if the health status is not significantly improved. Moreover, survivor bias should also be taken into consideration. The centenarians are a relatively healthy group of the older population (41). Centenarians with comorbidities are more tolerant of the adverse effects of diseases than others so that they can age well. Further studies are needed to validate our findings and better understand the mechanisms involved in this survival effect.

Several limitations need to be noted. First, this study is limited by its cross-sectional design, and no causal inference can be drawn. Longitudinal studies on depression and HRQoL will further clarify the predictive factors of HRQoL decline and provide potential targets for future interventions. Second, the depressive disorders were evaluated by GDS-15, not the clinical diagnosis. However, GDS-15 has been proven to be a stable assessment of depression and is commonly used for measuring depression in older people (42). Third, the results of self-reported questionnaires may be biased when the respondents are older adults with cognitive impairment. However, the same questions were asked to caregivers to ensure the authenticity of the information.

## Conclusion

This study demonstrated that depression is negatively related to HRQoL in Chinese centenarians. Elevated levels of GDS-15 score and depressive disorders are independent determinants of poor health states in the oldest-old. Especially, in centenarians without comorbidities, this association becomes more remarkable. However, large-scale prospective studies are needed to prove our findings and provide more information about the causal inference and internal mechanisms of this association.

## Data Availability Statement

The dataset used in this study can be obtained from the corresponding authors by a reasonable requests.

## Ethics Statement

The studies involving human participants were reviewed and approved by the Ethics Committee of the Hainan branch of the Chinese People's Liberation Army General Hospital. The patients/participants provided their written informed consent to participate in this study.

## Author Contributions

WJ, WC, ML, and YH contributed to the conception and design of the study. SW, YS, and JW managed the data and provided help in the data analysis. KH and SY performed the statistical analysis and wrote the first draft of the manuscript. All authors contributed to the study design, critically reviewed draft versions and provided important intellectual content during revisions, and accept accountability for the overall work.

## Conflict of Interest

The authors declare that the research was conducted in the absence of any commercial or financial relationships that could be construed as a potential conflict of interest.
